# Antibacterial surface engineering: bioinspiration from leaves to medical devices

**DOI:** 10.1039/d6lf00022c

**Published:** 2026-04-22

**Authors:** Dannielle M. Cox-Pridmore, Eleanor Mishra, Mark Webber, Sheng Qi

**Affiliations:** a School of Chemistry, Pharmacy and Pharmacology, Faculty of Science, University of East Anglia Norwich Research Park Norwich NR4 7TJ UK; b Quadram Institute Rosalind Franklin Road Norwich Research Park NR4 7UQ UK; c Centre for Microbial Interactions Norwich Research Park Norwich NR4 7UG UK; d Department of Respiratory Medicine, Norfolk & Norwich University Hospital Norwich NR4 7UY UK; e Faculty of Medicine & Health Sciences, University of East Anglia Norwich Research Park Norwich NR4 7TJ UK; f School of Pharmacy and Pharmaceutical Sciences, Institute of Systems, Molecular and Integrative Biology, University of Liverpool Liverpool L69 3GE UK sheng.qi@liverpool.ac.uk

## Abstract

Healthcare-acquired infections (HAIs) and the rise of antimicrobial resistance (AMR) are critical global health challenges, necessitating innovative solutions to combat pathogenic bacteria. Traditional approaches, such as antibiotics and chemical disinfectants, are increasingly ineffective due to the rapid evolution of resistant strains and their associated side effects and environmental impacts. The development of antimicrobial physical surface design strategies presents a promising alternative for reducing microbial colonisation and transmission. This review provides a comprehensive examination of antimicrobial surface geometries and topographies, focusing on physical surface strategies that prevent bacterial adhesion and biofilm formation. Inspired by naturally occurring structures such as insect wings and lotus leaves, these engineered surfaces employ nano- and micro-scale patterning to exert mechanical forces that disrupt microbial cells through membrane rupture or inhibit their attachment by limiting surface area for successful adhesion. We discuss key fabrication methods, mechanisms of action, material considerations, and clinical relevance, while also addressing challenges such as scalability, durability, and regulatory issues. By highlighting both the potential and limitations of physical surface modifications in healthcare environments, this review aims to inform future research and promote the integration of surface-based strategies in the design of next-generation medical devices and high-touch clinical surfaces.

## Abstract 2

Nano- and micro-structured, bioinspired surface architectures employ physical topographical cues to inhibit bacterial attachment, enabling fully chemical-free antimicrobial functionality. The graphical abstract image was created with BioRender.

## Graphical abstract

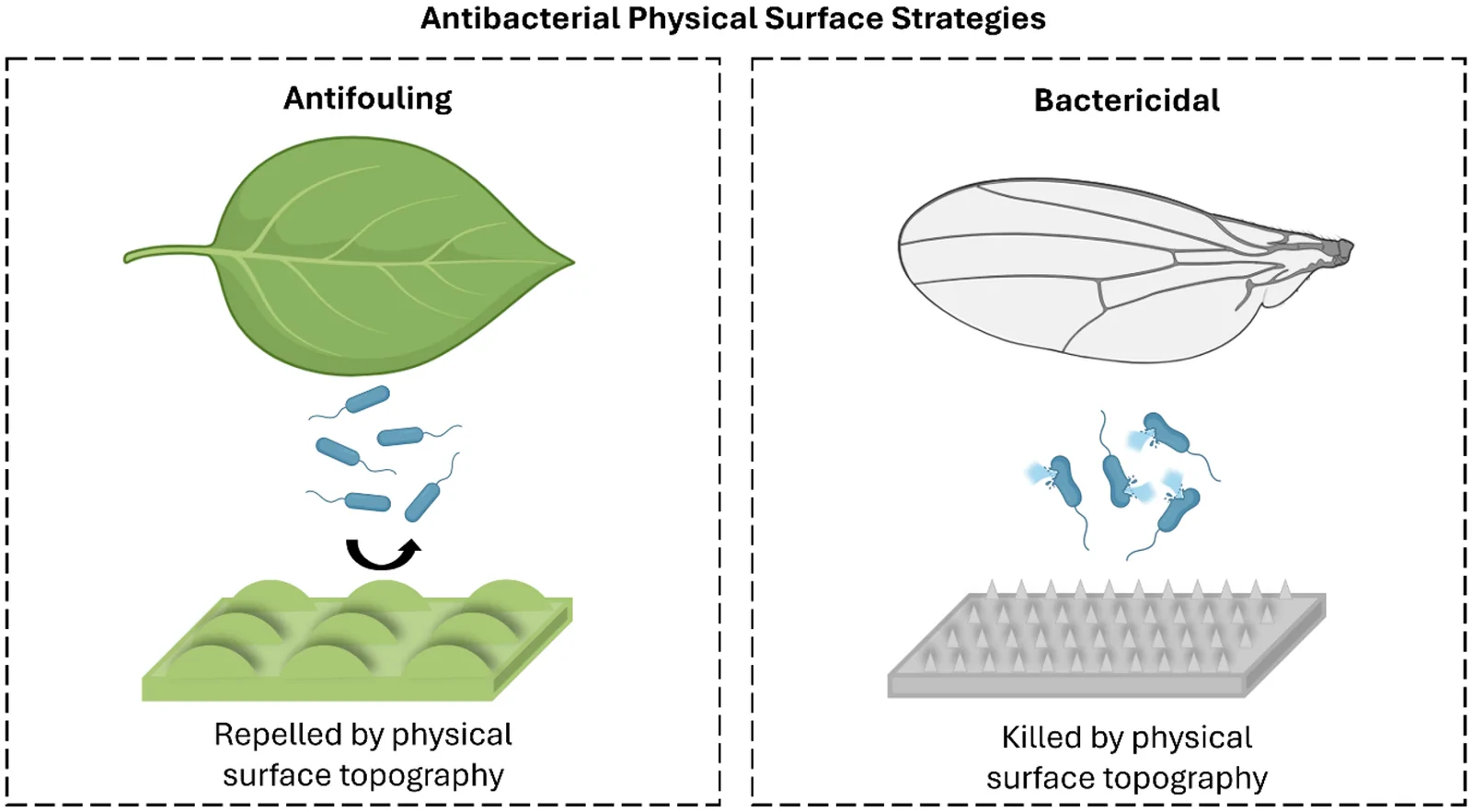

## 1. Introduction

### 1.1. Bacterial biofilms and infection

Bacteria are a family of single-cell microorganisms that play a crucial role in a wide array of biological processes, environmental cycles, and technological applications. Their remarkable ability to adapt to various environments, coupled with their metabolic diversity, makes them essential for maintaining ecological balance and supporting life on Earth. However, some bacteria are pathogenic, resulting in harmful infections, and therefore, are a major focus of medical research and public health efforts.^1–3^

**Fig. 1:**
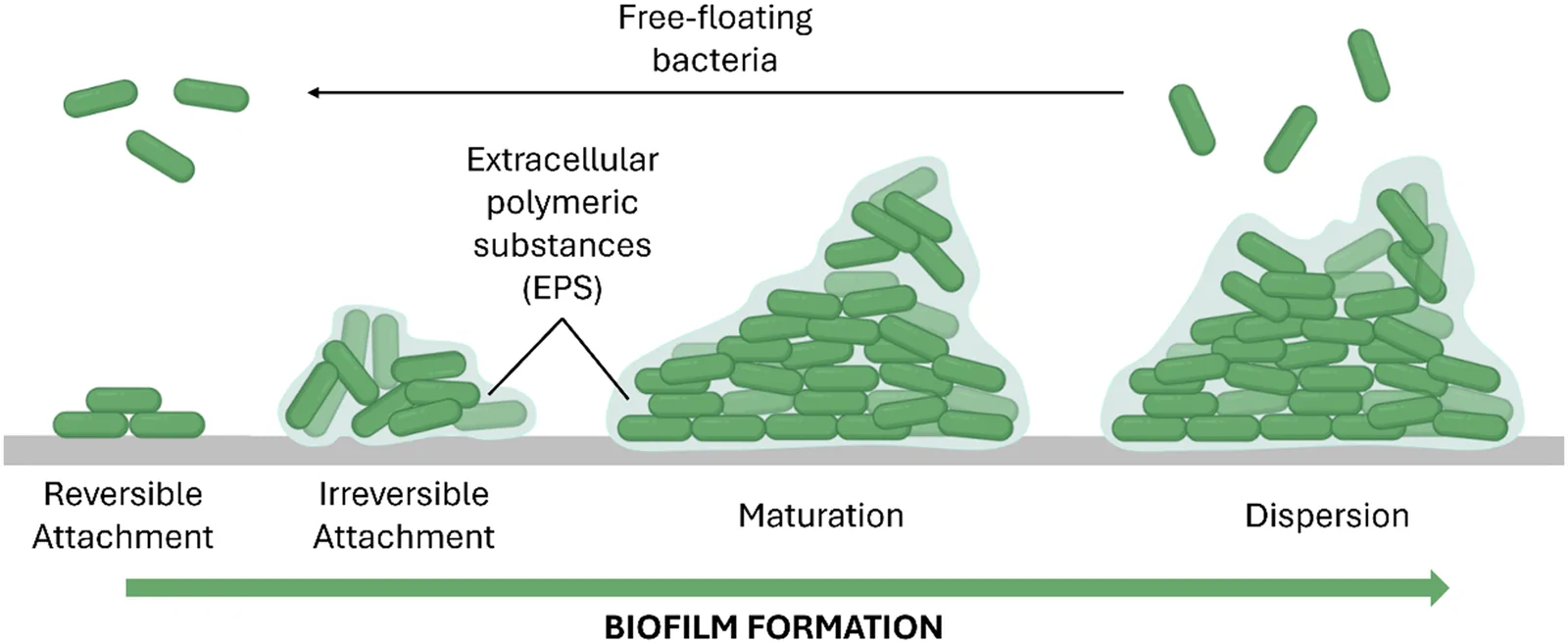
A schematic of bacterial adhesion and biofilm formation on a surface. Free-floating bacteria land on the surface, produce EPS, and become part of a resilient bacterial biofilm. Eventually, bacteria will disperse, resulting in the colonisation of new environments. Created using BioRender.

It is well established that bacteria can efficiently colonise surfaces and form a biofilm rather than dwelling in a planktonic state,^4,5^ with current estimates suggesting that up to 80% of bacteria reside in biofilms.^6^ A biofilm (Fig. 1) is a complex, structured community of bacteria encased in a self-produced matrix of extracellular polymeric substances (EPS), composed of polysaccharides, proteins, and nucleic acids,^7^ that adhere to living or inert surfaces such as medical devices, for example, catheters, implants, and prosthetics.^8–10^

Biofilm formation is a dynamic, multistage process that begins with the reversible attachment of planktonic (free-floating) microorganisms to the surface. This transitions to irreversible attachment, and bacteria begin to secrete EPS, forming a matrix that encases the cells and anchors them to the surface. As the biofilm grows and matures, microbial cells communicate with each other through quorum sensing to coordinate their activities, such as nutrient acquisition, metabolism, and defence mechanisms.^11^ The protective EPS barrier, reduced growth rates, high density of the bacterial cells, and the prevalence of persister cells within the biofilm enhance resistance to antibiotics, disinfectants, host immune defences, desiccation, and nutrient limitations, compared to planktonic bacteria. Eventually, environmental cues trigger the dispersion of cells from the biofilm, allowing them to colonise new surfaces and repeat the cycle. This entire process and the protective environment that the biofilm provides^12–14^ contributes to the persistence and chronicity of biofilm-associated infections,^15–18^ which are challenging to treat and eradicate.^19–24^

In healthcare settings, opportunistic pathogens frequently contribute to healthcare-associated infections (HAIs).^25,26^ HAIs are infections that patients acquire while receiving treatment for medical or surgical conditions within a healthcare setting, such as hospitals, outpatient clinics, nursing homes, and long-term care facilities. HAIs significantly increase medical care costs, length of hospital stays, and mortality rates. It is predicted that nearly 3.5 million people could lose their lives due to HAIs every year, up to 2050.^27^ Approximately 80% of HAIs can be attributed to biofilms, therefore, representing one of the greatest challenges in healthcare.^7,24,28^

### 1.2. Chemical approaches for managing bacteria and biofilms

Antibiotics are medications that are used to treat or prevent some types of bacterial infection. The extensive use of antibiotics is due to their broad range of effects, which include blocking the formation of bacterial cell walls, damaging cell membrane integrity, interfering with the production of nucleic acids and proteins, and disrupting essential metabolic processes. Therefore, it represents a first-choice drug for patients with an infection.^29–31^ However, due to the protective nature of biofilms, these infections are often resistant to conventional antibiotics.^14^ Therefore, the misuse and overuse of antibiotics have encouraged the development of antibiotic-resistant bacteria, further contributing to the development of chronic, life-threatening infections.^32,33^

Alongside antibiotics, disinfectants and chemical sanitisers are widely used to manage bacteria within healthcare settings to reduce HAI incidence. However, repeated use is practically and economically challenging. Moreover, there is growing evidence that extended interaction with such chemicals for sanitisation purposes (chlorine, hydrogen peroxide, sodium hypochlorite, ethanol) can lead to the enhanced probability of contracting long-term heart and lung diseases.^34–37^

Another chemical-based approach worthy of attention within healthcare settings is the application of metal-based surface coatings.^38,39^ These coatings incorporate metal ions (*e.g.*, silver, copper, zinc), metal oxides, or metal–organic frameworks,^40–42^ offering broad-spectrum antimicrobial activity through mechanisms such as reactive oxygen species (ROS) generation and bacterial membrane disruption.^26,43–46^ Despite their demonstrated antibacterial potential, several challenges hinder their widespread application. Ensuring stability under real-world conditions remains difficult, and the protective EPS matrix of biofilms can restrict ROS penetration. For example, silver-coated catheters are currently on the market with the ability to prevent infection for several days, but ultimately, result in biofilm formation, restricting long-term use.^47,48^ Moreover, coating efficacy is highly dependent on factors such as application method, environmental conditions, and surface cleanliness.^49^ The non-selective toxicity of metals and potential for metal ion leaching can lead to cytotoxicity and adverse host effects, posing significant barriers to clinical translation.^50,51^

Due to the nature of antibiotics, disinfectants, and metal-based coatings putting selective pressure on microbes, they result in increased risk of antimicrobial resistance (AMR) development.^39,52–55^ AMR poses a severe and escalating global health threat, pushing 24 million more people into extreme poverty in the next decade, and by 2050, will result in 10 million deaths annually and have an economic impact of $100 trillion.^56–58^ The spread of AMR endangers essential medical procedures, such as surgeries and chemotherapy, which rely on effective infection control; therefore, the development of new biofilm-prevention methods and rigorous stewardship of existing antimicrobials are crucial.

Given this tremendous concern, there is an increasing motivation for designing and developing antimicrobial surfaces that provide a sustainable approach to prevent biofilm formation and the spread of infections, while reducing our reliance on antibiotics and harsh chemical agents. Several reviews explore chemical-based approaches in more depth.^59–62^ This review focuses primarily on physical approaches to managing bacteria and biofilms.

## 2. Physical approaches for managing bacteria and biofilms

### 2.1. Background

Using surface topography is an innovative approach to achieving antibacterial effects without relying on chemical agents. By designing surfaces with specific patterns, such as ridges, spikes, or pillars, bacteria are prevented from adhering, colonising, and forming a stable connection to the surface to form a biofilm. Antibacterial surfaces can be broadly classified as either antibiofouling, which repels or resists bacterial attachment, or bactericidal, which actively kills bacteria. In some instances, an antibacterial surface may exhibit both antibiofouling and bactericidal characteristics.^59,63–68^ This strategy offers an alternative approach to traditional chemical-based methods to manage bacterial load. Such engineered topographies can be applied to medical devices, implants, and high-contact surfaces to maintain sterility and minimise infection risks within healthcare settings.

### 2.2. Nature-inspired biomimetic antibacterial physical strategies

Nature is an unexhausted source of inspiration for scientists and engineers, particularly for the development of biomimetic antibacterial and antifouling designs. Some natural surfaces have developed the ability to resist or prevent bacterial colonisation and biofilm formation.^69^ These surfaces include certain plant leaves, gecko feet, shark skin, insect wings, fish scales, and spider silk, which are frequently studied for their self-cleaning, low-adhesive, and superhydrophobic properties, many of which also confer antibacterial capabilities.^70,71^

**Fig. 2:**
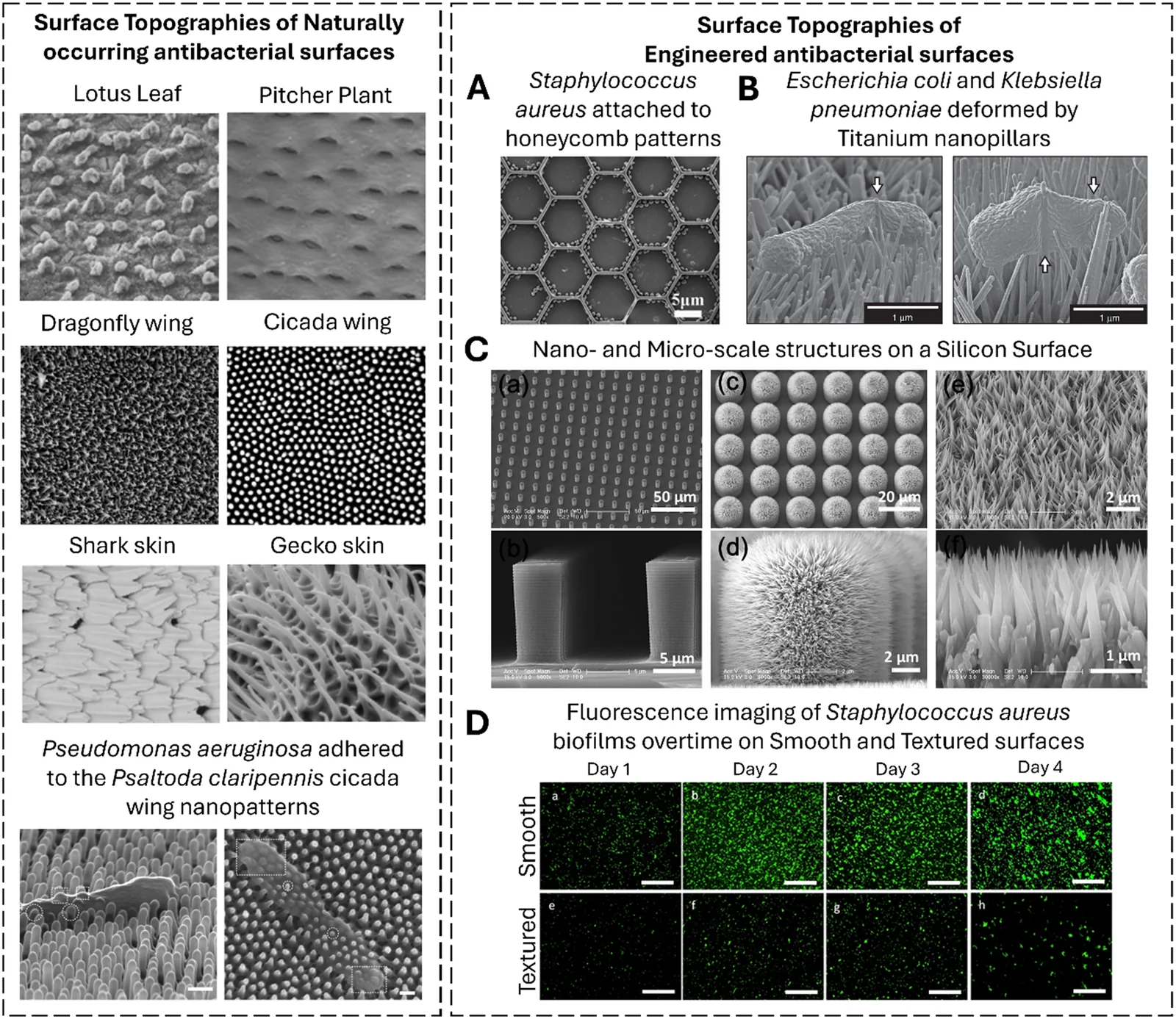
Naturally occurring and engineered surface topographies that demonstrate antibacterial behaviour. (Left) SEM images of naturally occuring surface features found on the lotus leaf,^110^ pitcher plant,^111^ dragonfly wing,^112^ cicada wing,^113^ shark skin,^73^ and gecko skin,^114^ including *P. aeruginosa* losing shape and turgor on cicada wing patterns, with outlines highlighting penetration and perturbation. Scale bars = 200 nm.^72^ (Right) SEM images of engineered surface features to demonstrate antibacterial behaviour. (A) 5 μm honeycomb-like patterns result in the random distribution and adhesion of *S. aureus* to the side walls.^115^ (B) *S. aureus* and *P. aeruginosa* (false coloured red) attached and ruptured by gold nanospikes.^116^ (C) Micro- and Nano-scale structures patterned on a silicon surface.^110^ (a) and (b) are microscale structures patterned surfaces; (c) and (d) are dual-scale structures patterned surface; (e) and (f) are pure nanoscale structure patterned surface ((a), (c) and (e) are images tilted at an angle of 45°, and (b), (d) and (f) are images at cross-sectional views). (D) Fluorescence images showing the reduction of *S. aureus* biofilms on textured surfaces (e–h) compared with smooth surfaces (a–d) after 1, 2, 3, and 7 days. Scale bar = 100 μm.^117^ Images were reprinted (adapted) with permission.^110^ Jiang *et al.*, (2020). Copyright 2020, Elsevier.^111^ Moran *et al.*, (2010), licensed under CC.^112^ Kamarajan *et al.*, (2020), licensed under CC.^113^ Pogodin *et al.*, (2013). Copyright 2013, Biophysical Society.^73^ Chien *et al.*, (2020). Copyright 2020, Elsevier.^114^ Watson *et al.*, (2015). Copyright 2015, Elsevier.^72^ Velic *et al.*, (2021). Copyright 2021, Elsevier.^115^ Yang *et al.*, (2015). Copyright 2015, Elsevier.^116^ Elbourne *et al.*, (2019), licensed under CC.^117^ Khursheed *et al.*, (2024), licensed under CC.

Several natural surfaces prevent microbial attachment through purely physical means, as shown in Table 1. For instance, cicada wings feature high-aspect-ratio nanopillar arrays that mechanically rupture bacterial membranes upon contact. As shown in Fig. 2, *P. aeruginosa* loses shape and turgor due to penetration from the wing features.^72^ Unlike chemical coatings, these surfaces rely on mechanical interactions to exert their bactericidal effects. Similarly, shark skin is composed of dermal denticles, which create a rough surface topography that reduces hydrodynamic drag and, therefore, microbial adhesion. The structured patterns alter local fluid dynamics and create a level of turbulence, potentially minimising the time microorganisms remain in contact with the surface and facilitating their removal.^73,74^ These principles have been commercially translated, most notably being Sharklet®, which replicates key features of shark skin topography to reduce bacterial attachment. Sharklet® is a synthetic surface fabricated *via* photolithography that mimics the ordered microtopography of dermal denticles using interlocking diamond-shaped riblets. The efficacy of Sharklet® surface patterns has been reviewed in more depth and demonstrates the varying efficacy depending on the microbial species (47–99% reductions),^63,75–78^ underscoring the inherent complexity of biological systems and the different ways different bacterial species will respond to the same surface.

**Table 1:** Examples of naturally occurring antibacterial physical surfaces

Surface	Features	Fabrication technique	Antibacterial activity	Ref.
Lotus leaf (*Nelumbo nucifera*)	Micro-papillae, 100 μm height and 100 μm spacing. Wax crystalloids, 1–5 μm in height. Tabular and convex papillae, 30–50 μm length and 10–30 μm width. Superhydrophobic (WCA = 162°)	Natural	Antibiofouling	118–120
Taro leaf (*Colocasia esculenta*)	Nano (<0.5 μm), micro (>0.5–10 μm) and macro topographies (>10 μm). Superhydrophobic (WCA = 164°)	Natural	Antibiofouling	118, 121, 122
Shark skin (*Carcharodon carcharias*)	Dermal denticles, 26.8 μm height, 99.5 μm spacing	Natural	Antibiofouling	123
Shark skin (*Centrophorus granulosus*)	Dermal denticles, 15.8 μm height, 136.8 μm spacing	Natural	Antibiofouling	123
Cicadas	*Megapomponia intermedia*, pillars, 241 nm height, 156 nm diameter, 165 nm spacing	Natural	Bactericidal against Gram-negative bacteria (*Pseudomonas fluorescens*)	124
	*Ayuthia spectabile*, pillars, 182 nm height, 207 nm diameter, 251 nm spacing			
	*Cryptotympana aguila*, pillars, 182 nm height, 159 nm diameter, 187 nm spacing			
Cicada wing (*Psaltoda claripennis*)	Nanoneedles, 200 nm height, 170 nm spacing. Superhydrophobic (WCA = 158–159°)	Natural	Bactericidal against Gram-negative bacteria (*Pseudomonas aeruginosa*)	67, 125
Gecko skin (*Lucasium* sp.)	Spinule structures, 3000 nm height, 500 nm spacing, tip radius of curvature <20 nm	Natural	Bactericidal against Gram-negative bacteria (*Porphyromonas gingivalis*)	114
	Superhydrophobic (WCA = 150–155°)			
Dragonfly wing (*Diplacodes bipunctata*)	Nanopillars, 240 nm height, 200 nm spacing. Superhydrophobic (WCA = 153°)	Natural	Bactericidal against Gram-positive (*Bacillus subtilis*) and Gram-negative (*Pseudomonas aeruginosa*) bacteria	126–128

It is important to note that the experimental methods to understand bacterial behaviour vary, reflecting a lack of standardisation and methodological uniformity across studies,^79^ as shown in Tables 2 and 3. Commonly used methods to assess the antibacterial activity of surfaces include viability staining with fluorescent dyes such as SYTO 9 and propidium iodide (PI), colony-forming unit (CFU) counts, and imaging techniques such as confocal laser scanning microscopy (CLSM), fluorescence microscopy, and scanning electron microscopy (SEM). These approaches can provide complementary information, but each technique has notable limitations. CFU counts enable quantification of viable, culturable bacteria; however, this method often underestimates bacterial survival due to the presence of viable but non-culturable (VBNC) populations or incomplete detachment of biofilms from surfaces.^80–83^

**Table 2:** Examples of bactericidal physical surface topographies. *Material chemistry may synergise with surface structures to demonstrate antibacterial effect. Propidium iodide (PI). Colony forming unit (CFU). Confocal laser scanning microscopy (CLSM). Scanning electron microscopy (SEM). Atomic force microscopy (AFM)

Surface	Features	Fabrication technique	Antibacterial activity testing methods	Bactericidal efficacy	Ref.
Black silicon	Nanograss, 500 nm height, 20–80 nm diameter. Hydrophilic (WCA = 80°)	Reactive ion etching	SYTO 9/PI	Effective against Gram-positive (*Bacillus subtilis*) and Gram-negative (*Pseudomonas aeruginosa*) bacteria	126
			CLSM		
			CFU counts		

Black silicon	Nanograss, 4 μm height, 220 nm diameter	Deep reactive ion etching	SYTO 9/PI	Effective against Gram-positive (*Staphylococcus aureus*) and Gram-negative (*Escherichia coli*) bacteria	140
	Superhydrophobic (WCA = 154°)		Fluorescence microscopy		
			CFU counts		

Black silicon	Nanopillars 280 nm height, superhydrophilic (WCA = 8.4° ± 2.1°)	Plasma etching	SYTO 9/PI	Effective against Gram-positive (*Staphylococcus aureus*) and Gram-negative (*Pseudomonas aeruginosa*) bacteria	141
	430 nm height, hydrophilic (WCA = 12.4° ± 1.2°)		CLSM		
			SEM		
	610 nm height, superhydrophilic (WCA = 9.9° ± 1.0°)				

Diamond-coated* black silicon	Nanoneedles, 0.5–1 μm height (short needle) and 15–20 μm height (long needle)	Plasma etching and vapour deposition	SYTO 9/PI	Effective against Gram-negative (*Pseudomonas aeruginosa*) bacteria	142
			Fluorescence microscopy		
			SEM		

Cicada-inspired diamond* surface	Nanocones with sharp tips, 3–5 μm height, 10–40 nm tip diameter, 350 nm–1.2 μm nanocone width	Microwave plasma chemical vapour deposition and reactive ion etching	SYTO 9/PI	Effective against Gram-negative (*Pseudomonas aeruginosa*) bacteria	143
			Fluorescence microscopy		
			SEM		

Dragonfly-inspired titanium (Ti)* nanopatterned array	Nanowires, average diameter 40.3 nm. Hydrophilic (WCA = 73°)	Hydrothermal etching	SYTO 9/PI	Effective against Gram-negative (*Pseudomonas aeruginosa*) bacteria	144
			CLSM		
			SEM		

Cicada wing-inspired Ti* columns	Nanocolumns, 478 ± 6 nm height. Nano-sized grains, 33 ± 7 nm width. 26 ± 3 nm (*R*_q_) surface roughness	Glancing angle sputter deposition	SYTO 9/PI	Effective against Gram-negative (*Escherichia coli*) bacteria	145
	Hydrophilic (WCA = 41.48° ± 0.83°)		Fluorescence microscopy		
			SEM		

Ti alloy* surface	Nanospikes, 2 μm height, 10 nm diameter, 2 μm spacing	Anodisation	SYTO 9/PI	Effective against Gram-positive (*Staphylococcus aureus*) bacteria	146
			Fluorescence microscopy		
			SEM		

Ti alloy* surface	Nanospikes, 20 nm diameter	Thermal oxidation	SYTO 9/PI	Effective against Gram-negative (*Escherichia coli*) bacteria	147
			Fluorescence microscopy		
			PrestoBlue®		

Cicada-inspired Titania* nanowire arrays	Nanowires	Hydrothermal process	SYTO 9/PI	Effective against motile bacteria (*Pseudomonas aeruginosa*, *Escherichia coli* and *Bacillus subtilis*)	148
	Brush type: 100 nm diameter		Fluorescence microscopy		
	Niche type: 10–15 μm diameter		PrestoBlue®		
			SEM		

Titania* micro-nanopillar arrays	Pillar array sizing 20 μm, 10 μm, 5 μm, 2 μm, 1.4 μm, 1.2 μm, 1.0 μm, 0.8 μm, and 0.6 μm	Photolithography, RF magnetron sputtering and thermal oxidation	SYTO 9/PI	Effective against Gram-positive (*Staphylococcus aureus*) and Gram-negative (*Escherichia coli*) bacteria	149
	Hydrophilic (WCA < 90°)		CLSM		
			SEM		

Aluminium (Al 6063 alloy) nanostructured surface	Etching time: 0.5 h, 69.9 ± 5.3 nm (*R*_rms_), hydrophilic (WCA = 28.2° ± 3.3°)	Wet chemical etching	SYTO 9/PI	Effective against Gram-positive (*Staphylococcus aureus*) and Gram-negative (*Pseudomonas aeruginosa*) bacteria	150
	1 h, 133.6 ± 36.1 nm (*R*_rms_), hydrophilic (WCA = 21.8° ± 4.7°)		Fluorescence microscopy		
	3 h, 995 ± 114.7 nm (*R*_rms_), hydrophilic (WCA = 17.7° ± 4.3°)				

Cicada wing-inspired PMMA nanopatterned surface	Nanopillars, 200–300 nm height, 70–215 nm diameter, 130–380 nm spacing	Nanoimprint lithography	SYTO 9/PI	Effective against Gram-negative (*Escherichia coli*) bacteria	151
			Fluorescence microscopy		
			CFU counts		
			SEM		
			AFM		

Poly-l-lactic acid (PLLA) surface	Cylindrical micropillars, 40 μm height, 40 μm width decorated with nanostructures. Hydrophobic (WCA >100°)	Nanoimprint lithography	ISO 207043:2007	Effective against Gram-positive (*Staphylococcus aureus*) and Gram-negative (*Escherichia coli*) bacteria	152
			CFU counts		
			SEM		

Polycarbonate surface	Nanopillars arrays, 143–408 nm height, 100–313.9 nm spacing	Nanoporous anodic aluminium oxide (AAO) template-assisted hot embossing and wet etching	SYTO 9/PI	Effective against Gram-negative (*Escherichia coli*) bacteria	153
	Hydrophobic		Fluorescence microscopy		
			SEM		

Gecko skin-inspired nano-spinules	Spinules, 1–4 μm height, 600 nm spacing. Hydrophobic (WCA = 134°)	Nano-templating	SYTO 9/TO-PRO-3	Effective against Gram-positive bacteria (*lactobacillus*)	154
			Laser confocal microscopy		
			SEM		

Cicada wing-inspired gold (Au)* nanopatterns	Nanopillars (100 nm height, 50 nm diameter)	Electrodeposition and plasma etching	SYTO 9/PI	Effective against Gram-positive (*Staphylococcus aureus*) bacteria	155
	Nanorings (100 nm height, 100–200 nm diameter)		Fluorescence microscopy		
	Nanonuggets (100 nm height, 100–200 diameter)		SEM		

PDMS or PMMA nanopatterns	Moth-eye nanocones, 250 nm diameter at the base and 80 nm on the top, 350 nm height and 250 nm spacing	PDMS-soft lithography	SYTO 9/PI	Effective against Gram-positive (*Staphylococcus aureus*) and Gram-negative (*Escherichia coli*) bacteria	156
		PMMA-thermal nanoimprint lithography	Fluorescence microscopy		
			SEM		

**Table 3:** Examples of antifouling physical surface topographies. *Material chemistry may synergise with surface structures to demonstrate antibacterial effect. Propidium iodide (PI). Confocal laser scanning microscopy (CLSM). Scanning electron microscopy (SEM). Colony forming unit (CFU). Green fluorescent protein (GFP)

Surface	Features	Fabrication technique	Antibacterial activity testing methods	Antifouling efficacy	Ref.
Silicon micropillar arrays	Various sizings: 20 μm, 10 μm, 5 μm, 2 μm, 1.4 μm, 1.2 μm, 1.0 μm, 0.8 μm, and 0.6 μm	Photolithography and dry etching	SYTO 9/PI	Effective against Gram-positive (*Staphylococcus aureus*) and Gram-negative (*Escherichia coli*) bacteria	180
	Hydrophilic (WCA < 90°)		CLSM		
			SEM		

Silicon honeycomb pattern with micro-size pores	Pore sizes: 0.5 μm, hydrophilic (WCA = 70° ± 4°)	Photolithography and deep reactive ion etching	SYTO 9	Effective against Gram-positive (*Staphylococcus aureus*) and Gram-negative (*Escherichia coli*) bacteria	115
	1 μm, hydrophobic (WCA = 100° ± 2°)		CLSM		
	3 μm, hydrophobic (WCA = 118° ± 1°)		SEM		
	5 μm, hydrophilic (WCA = 84° ± 2°)				
	10 μm, hydrophilic (WCA = 80° ± 2°)				

Lotus leaf-inspired Ti* surface	Micro-grains (10–20 μm), Nano-undulations (<200 nm). Superhydrophobic (WCA = 166°)	Femtosecond laser irradiation	SYTO 17/Concanavalin A Alex Fluor 488	Effective against Gram-positive (*Staphylococcus aureus*) and Gram-negative (*Pseudomonas aeruginosa*) bacteria	181
			CLSM		
			SEM		

Ti* mesopores	Pores, 20–25 nm diameter, ∼17 nm (*R*_rms_) surface roughness	Chemical oxidation	SEM	Effective against Gram-positive (*Staphylococcus aureus*) and Gram-negative (*Escherichia coli*) bacteria	182

Lotus leaf-inspired Ti alloy* surface	Various surface textures (390 nm, 660 nm, and 1.1 μm)	Laser texturing	SEM	Effective against gram negative (*Escherichia coli*) bacteria	122

Nanostructured PMMA film	Nanopores, 460 nm depth, 300 nm spacing	Nanoimprint lithography	GFP-expression	Effective against Gram-negative (*Pseudomonas aeruginosa* and *Escherichia coli*) bacteria	183
	Hydrophobic (WCA = 114.5°)		Fluorescence microscopy		

Shark skin-replicated PMMA microstructure	Tail abdomen denticles, 164.5 μm height, 85.7 μm width, 118.8 denticles per mm^2^. Hydrophobic (WCA = 115.8°)	Polymer imprinting	Crystal violet	Effective against Gram-positive (*Staphylococcus aureus*) and Gram-negative (*Escherichia coli*) bacteria	73
	Pectoral fin denticles, 166.2 μm height, 69.8 μm width, 83.3 denticles per mm^2^. Hydrophobic (WCA = 105.6°)				

PDMS micropatterned surface	Square features, 6 μm height, 4 my spacing	Soft lithography	Quantified manually	Effective against Gram-positive (*Staphylococcus aureus* and *Staphylococcus epidermidis*) bacteria	184
	Circular features, 3 μm diameter, 2 μm spacing. Ridges, 2 μm width and 3 μm-wide channels. Hydrophobic (WCA = 108.7°)		SEM		

Polystyrene spheres	630–1550 nm monodisperse spheres	Colloidal crystal fabrication	Light microscopy	Effective against Gram-negative (*Pseudomonas aeruginosa*) bacteria	157
			SEM		

Polypropylene (PP) films	Micropillars, 10 μm height, 10 μm diameter, 23 μm spacing	Nanoimprint lithography and reactive ion etching	Touch transfer assay	Effective against Gram -ositive (*Staphylococcus aureus*) and Gram-negative (*Escherichia coli*) bacteria	108
	Nanospikes, 2.2 μm height, 300 nm base diameter. Hydrophobic (WCA >100°)		CFU count		

Shark skin- mimicked chitosan*/graphene oxide* membranes	Topographies from the skin of a basking shark (*Cetorhinus maximus*)	Negative Mold	ISO 22196	Effective against Gram-positive (*Staphylococcus aureus*) and Gram-negative (*Escherichia coli*) bacteria	185
			CFU count		
			MTT assay		
			SEM		

Nanostructure sutures	Lamella structure, 500 nm length, 100 nm thickness	Plasma etching	SYTO 9/PI	Effective against Gram-negative (*Escherichia coli*) bacteria	186
			Fluorescence microscopy		
			CFU counts		
			SEM		

Structured polystyrene surface	Lamella structures, 0.47 μm width, 2 μm spacing	Direct laser interference patterning	CFU counts	Effective against Gram-positive (*Staphylococcus aureus*) bacteria	187
			SEM		

Fluorescence-based assays and microscopy allow visualisation of cell viability and spatial distribution. CLSM in particular can provide three-dimensional information on biofilm structure, whereas SEM offers high-resolution imaging of bacterial morphology and surface interactions.^84–86^ Fluorescent staining approaches can be influenced by staining conditions such as dye concentration, incubation time, and biofilm thickness, which may affect dye penetration and lead to misinterpretation of viability.^87–90^ Microscopy-based techniques rely on image processing or fluorescence intensity measurements that can vary depending on imaging parameters. In addition, SEM requires extensive sample preparation, which may alter biofilm structure.^91^ A further challenge across these methods is the lack of standardised protocols for dye concentrations, incubation conditions, and quantification approaches, making comparisons between studies difficult. Consequently, while these techniques are widely used to evaluate antibacterial surfaces, careful methodological control and the combination of multiple complementary assays are often necessary to obtain reliable and reproducible assessments of antibacterial performance.

Biomimetic replication of features seen in nature has led to promising engineered surfaces. For example, Kumar and Bhardwaj (2020) replicated the hierarchical structure of the taro leaf using photolithography, achieving static contact angles up to 148° by mimicking the dual-scale hexagonal and flake-like microstructures.^92^ However, traditional fabrication methods, such as photolithography, make the whole process costly and time-consuming, with limited scalability. Therefore, there has been a lack of commercialisation of such surfaces. However, the research and these examples underscore the effectiveness of biologically inspired surface topographies in resisting bacterial colonisation and biofilm development.

For antibacterial surface topographies to achieve translational and commercial impact, scalable and high-throughput manufacturing techniques need to be considered and used to produce nano- and micro-scale features reliably and reproducibly over large areas, with relevant materials, and on clinically relevant substrates. Roll-to-roll nanoimprint lithography offers significant promise, enabling continuous pattern transfer onto polymer films at industrial speeds with high reliability and relatively low cost per unit. In combination with nanocoining, the manufacturing can also overcome the high cost associated with a master mould. This approach is particularly attractive for disposable medical products, wound dressings, and device surfaces, where large-area replication and throughput are critical.^93–95^ Similarly, laser-based methods, such as laser-induced periodic surface structures (LIPSS), provide a single-step method for creating features directly onto metallic or polymeric surfaces. LIPSS is especially relevant for orthopaedic implants, surgical tools, and catheter components, as it can be applied to complex geometries and hard materials while allowing spatial control of patterning.^96–99^ Other scalable approaches, including injection moulding with textured moulds,^100,101^ hot embossing,^102,103^ and reactive ion etching,^104,105^ also present industrially viable routes, particularly when integrated into existing medical device manufacturing pipelines.

However, for these techniques to be clinically feasible, they must demonstrate not only pattern uniformity and reliability, but also compatibility with regulatory requirements, sterilisation processes, mechanical durability, and cost constraints. Moreover, reproducibility across large production batches and the ability to pattern three-dimensional, curved, or flexible substrates remain practical challenges. A deeper consideration of manufacturing scalability, including tooling lifetime, cycle time, material compatibility, and quality control metrology, will therefore be essential to bridge the gap between laboratory proof of concept research and commercially available antibacterial medical devices and healthcare equipment. Detailed reviews of fabrication strategies and manufacturing techniques are available.^106,107^

As mentioned, for physical antibacterial topographies to achieve translational feasibility, they must demonstrate durable antimicrobial efficacy alongside mechanical and chemical stability under real-world conditions. In clinical and industrial settings, the topographies would be routinely exposed to extensive cleaning procedures, repeated sterilisation cycles, and frictional wear. Francone *et al.*, (2021) fabricated patterned polypropylene and conducted washability tests mimicking hospital-specific cleaning protocols, which demonstrated that the features of the surface, such as topography, water contact angle, and gloss, remained unchanged despite intensive physical and chemical cleaning procedures over time.^108^ This demonstrates promise for the application of nano- and micro-patterning within clinical settings. Theoretically, if the patterns are not damaged, the surface can be reused indefinitely when cleaned.^109^ However, from this literature review, it is seen that many studies show high initial promise but rarely test the durability of the features against wear, fouling, and bacterial resistance, with more research needing to be conducted within this area. Therefore, the rational design of antibacterial surface topographies must integrate considerations of mechanical durability, chemical resistance, and wear stability alongside antimicrobial performance to ensure function throughout the intended lifespan of the surface.

### 2.3. Bactericidal physical surface topographies

Bactericidal surface topographies are designed to actively kill bacteria through mechanical forces. These surfaces often feature nanoscale structures such as spikes, pillars, or sharp ridges that can promote adhesion and then stretch, puncture, or rupture bacterial cell membranes upon contact, as they grow, or during movement, and if the applied force exceeds their elasticity, leading to bacterial lysis and death. This deformation-stretching-torn apart effect has been observed in various engineered surfaces, including biomimetic designs inspired by cicada and damselfly wings.^113,129,130^ The ability of a bacterium to rupture due to a nanopatterned surface may also depend on the maturation stage of the cell, as shown by Truong *et al.*, (2017). The nanopillars present on the wings of the damselfly (*Calopteryx haemorrhoidalis*) exhibited the highest bactericidal activity in the early (1 hour) and late (24 hour) stationary phase of *Staphylococcus aureus* and *Pseudomonas aeruginosa*, resulting in 89.7 and 61.3% as 97.9 and 97.1% dead cells upon surface contact, respectively.^131^ These findings highlight that strong bacterial adherence to the nanopatterned surface is critical for effective mechano-bactericidal activity. High-aspect-ratio structures are particularly important for promoting initial bacterial attachment, which facilitates subsequent membrane rupture, for example, hierarchical structures like two-tiered micropillar arrays.^129^ The bactericidal performance of such surfaces is strongly influenced by structural characteristics, including pillar height, diameter, density, and spatial arrangement. Denser and smaller nanopillar arrays have been found to be more effective against both Gram-negative and Gram-positive bacteria compared to larger, widely spaced configurations,^132^ because they need to promote adhesion to be able to demonstrate their killing effect.

Other characteristics of bacteria, such as their motility, morphology, and size, can influence their interactions with nanopatterned surfaces. Highly motile bacteria experience greater membrane deformation due to movement across the surface, leading to increased cell rupture.^133^ Morphological differences, such as being coccoid or rod-shaped, can impact their interactions with the same surface. The relative dimensions of bacterial cells and surface patterns are also critical. Bactericidal efficiency is enhanced when cell size exceeds the spacing between nanopatterns, allowing for direct penetration and rupture. Conversely, smaller bacterial cells may stretch or compress against the sidewalls of nanostructures, resulting in different mechanical stress responses. Kelleher *et al.*, (2016) demonstrated that surfaces with tighter nanopillar spacing and smaller feature sizes showed superior bactericidal activity compared to those with lower density and larger features.^124^ Modaresifar *et al.*, (2019) systematically reviewed bactericidal nanopatterning and reported that most design parameters tend to fall within specific ranges: feature height between 100–1000 nm, diameter between 10–300 nm, and spacing less than 500 nm.^134^ A variety of materials and surfaces have been explored to demonstrate bactericidal activity, for example, black silicon is widely used, which is regular silicon etched to produce nanoscale markings that make it appear black due to trapping of light.^135^ In order to show the breadth of research in this area, Table 2 is generated to summary the materials used, feature dimensions, fabrication techniques, bactericidal activity, and how it is evaluated. It explores several examples of bactericidal surface patterns with their respective features and what they are effective against.

A challenge in the field of antibacterial surface topographies is the lack of standardisation and methodological uniformity across existing studies, which significantly limits comparison, *meta*-analysis, and reproducibility. A critical evaluation by Redfern *et al.*, (2024) revealed substantial variation in growth conditions, controls, and analytical measurements of biofilms performed by different research groups.^79^ Standardisation of microbiological practice is inherently difficult due to differences in equipment, technical expertise, and laboratory resources across institutions. Furthermore, biofilm science itself remains an evolving field, with ongoing debate regarding the most appropriate methods to grow, control, quantify, and analyse bacteria and biofilms.

In addition, there is often an inadequate characterisation of the topography. For example, relying on parameters like average roughness, which does not describe the dimensions of the pattern itself.^136^ Surfaces with identical roughness values may have different heights, widths, spacing, and shapes, which also significantly impact how bacteria interact with the surface. Researchers must provide sufficient details of topographical descriptors such as feature height, width, spacing, shape, surface chemistry, *etc.*, as well as experimental conditions such as bacterial strain selection, growth phase, inoculum density, media composition, incubation duration, and environmental parameters, in addition to reasoning behind quantitative outcome measures.

Interactions at the bacteria–surface interface are complex, dynamic, and heavily dependent on specific environmental conditions. There is no ‘one-size-fits-all’ solution with patterned surfaces, and the features need to be tailored depending on the bacterial species and the environment it will be experiencing. The optimisation of pattern design, including parameters such as feature density, stiffness, and geometry, is therefore needed to enhance the antibacterial surface performance for different bacterial species.^137–139^

Optimal topographical design is likely to be context specific. A micro- or nano-scale pattern that reduces bacterial colonisation on a dry, high-touch hospital surface may not perform equivalently on an implanted medical device, where continuous fluid flow, immune interactions, and long-term mechanical stresses alter bacterial behaviour. Therefore, proposed best practices should not only encourage comprehensive reporting of surface parameters, microbiological conditions, and quantitative antibacterial measurements, but also require clear justification of experimental models in relation to the intended end-use environment. Establishing such a structured reporting framework would enhance reproducibility, enable meaningful comparisons, and provide a more rigorous foundation for translating antibacterial topographic surfaces into clinically and industrially relevant applications.

### 2.4. Antibiofouling physical surface topographies

Antifouling surface topographies are engineered to prevent bacterial adhesion and colonisation rather than directly killing the cells. The surface features often create an energetically unfavourable environment for microbial attachment, or geometries that physically limit the ability of bacteria to establish stable contact. Table 3 explores some examples of antibiofouling surfaces along with the species they are reported to be successful against. As shown, these features range from the nano- to the micro-scale; however, the broader literature provides a fragmented, often incomplete, and potentially contradictory view of antifouling features and what works best. For example, Kargar *et al.*, (2014) reported that larger spherical features delayed biofilm formation compared to smaller ones.^157^ Yet literature often recommends smaller topographic sizes to inhibit biofilm formation.^158,159^

Mok *et al.*, (2019) showed that concave periodic boundary geometries limited *Escherichia coli* accumulation,^160^ while Gu *et al.*, (2017) demonstrated that hexagonally arranged topographies with optimised spacing disrupt intercellular interactions and limit biofilm formation.^161^ In another study, Gu *et al.*, (2016) further demonstrated that bacterial orientation tends to align perpendicularly to linear patterns, with more random attachment occurring as line widths increase and approach planar surfaces.^162^ Ge *et al.*, (2019) attributed antibacterial efficacy (*e.g.*, 62% reduction in *S. aureus* adhesion, and 73% reduction in *E. coli* adhesion) to the synergistic effects of reduced contact area and spatial confinement offered by pillar-based topographies.^149^ However, it is important to note that this group used Titanium oxide, which is inherently antibacterial; therefore, potential effects may not solely be due to physical approaches. In Tables 2 and 3, an annotation (*) has been made where groups have used materials that may have particular chemistries that may synergise with surface structures to demonstrate an antibacterial effect; the result is not entirely through physical means. It is important to note that a major benefit of antibacterial activity through physical mechanisms induced by nano- and micro-scale topographies is that they do not exert selective pressure for resistance development like conventional antibiotics and other chemical-based methods do.^163,164^ However, long-term efficacy and other potential impacts require further study.^164,165^

Antifouling topographies reduce the likelihood of biofilm formation by making it difficult for bacteria to remain anchored long enough to multiply and produce EPS, through the alteration of surface properties such as roughness, contact forces, wettability, shear stress, and fluid flow. Increased surface roughness reduces bacterial adhesion by limiting available adhesion points. The reduction in contact area also impacts surface charge accumulation and distribution, therefore, altering the attractive and repulsive forces between the bacteria and the surface.^138,166,167^ Effectively, repelling the bacteria and reducing bacterial attachment.

Wettability also plays a crucial role in antibiofouling.^168^ Superhydrophobic surfaces (water contact angle >150°) trap air pockets, creating a barrier against bacterial attachment, while superhydrophilic surfaces (water contact angle <5°) form a continuous water layer that prevents direct contact with bacteria.^169^ Hierarchical structures, such as those found on lotus leaves, exhibit extreme wettability due to the combination of micro-scale bumps and nanoscale hairs. These structures allow water droplets to bead up and roll off easily, effectively preventing bacterial colonisation through self-cleaning mechanisms. A study developing titanium-based lotus leaf-like surfaces found that bacteria were unable to adhere due to trapped air nano- and micro-bubbles, which minimised contact and imparted antifouling properties.^170^ Ellinas *et al.*, (2017) reported that a superhydrophobic polymethyl methacrylate (PMMA) surface with a water contact angle (WCA) above 155° exhibited prolonged bacterial repellence against *Synechococcus* sp. for up to 72 hours and minimised adhesion for four days.^171^ However, superhydrophobicity typically diminishes during prolonged immersion in liquid due to air layer depletion. Therefore, careful control of feature size and surface design is essential to sustain the air layer and preserve antibacterial efficacy, and the design must be tailored to the specific environment in which it will be applied to achieve the greatest effect.

While a substantial body of literature explores surface topographies influencing bacterial attachment and biofilm formation, considerably fewer studies focus on elucidating the underlying mechanisms responsible for these effects. A deeper mechanistic understanding of how and why specific surface features inhibit biofilm development is therefore required. One mechanism of action might be the interruption of cellular signalling. As previously mentioned, quorum sensing is a form of bacterial cell-to-cell communication.^11,172^ Disrupting signalling capabilities may, in turn, disrupt stable, long-term contact of bacteria with a surface. Recent work with *P. aeruginosa* has explored the influence of nanotopographies on quorum-sensing molecule (QSM) production and their ability to reduce major virulence pathways.^173^ In addition, Romero *et al.*, (2025) were able to explore that microtopographies resulted in quorum-sensing-mediated autolubrication that inhibits early-stage *P. aeruginosa* biofilm formation. It appears that the spatial restrictions of the topographies result in the production of lubricating rhamnolipids that prevent irreversible bacterial adhesion to the surface.^174^

Other potential methods for topographies to induce antibacterial effects include altering surface contact area, therefore reducing stable attachment and disrupting the spatial organisation required for biofilm formation. It is also seen that topographies can influence hydrodynamic forces in the near-surface environment, increasing velocity, which can disrupt initial bacterial settlement. It is possible that the presence of patterns on the surface and their effect on the surrounding environment would influence quorum sensing by limiting the effective diffusion and local accumulation of autoinducers, preventing them from reaching the threshold concentrations necessary to trigger coordinated gene expression required for stable attachment and biofilm formation.^175–178^ Such mechanistic insights will enable a more comprehensive interpretation of existing results, provide further validation of observed antimicrobial performance, and support the rational design and broader application of topographically engineered surfaces across diverse environments.

By optimising surface topography, including feature dimensions, spacing, and wettability characteristics, antibiofouling surfaces can effectively minimise bacterial adhesion and biofilm development, providing a promising strategy for antimicrobial surface design. Recently, the use of machine learning (ML)^174^ and artificial intelligence (AI)^179^ to determine the best antimicrobial shapes and features promises accelerated discovery. Yet these methods are limited by the complexity of biological systems, which are difficult to emulate, and the lack of standardised data on topographies and their performance, which can reduce the model's accuracy. It is essential to consider the specific type of bacteria being targeted, as well as the environmental conditions in which the surface will be applied, since these factors strongly influence the effectiveness of the design. Because they focus on prevention and reduction rather than destruction, antibiofouling topographies are especially valuable in applications where long-term resistance to biofilm growth is needed, and they can be combined with chemical coatings or bactericidal features to provide more comprehensive antibacterial protection.

## 3. Next-generation dynamic surface topography

**Fig. 3:**
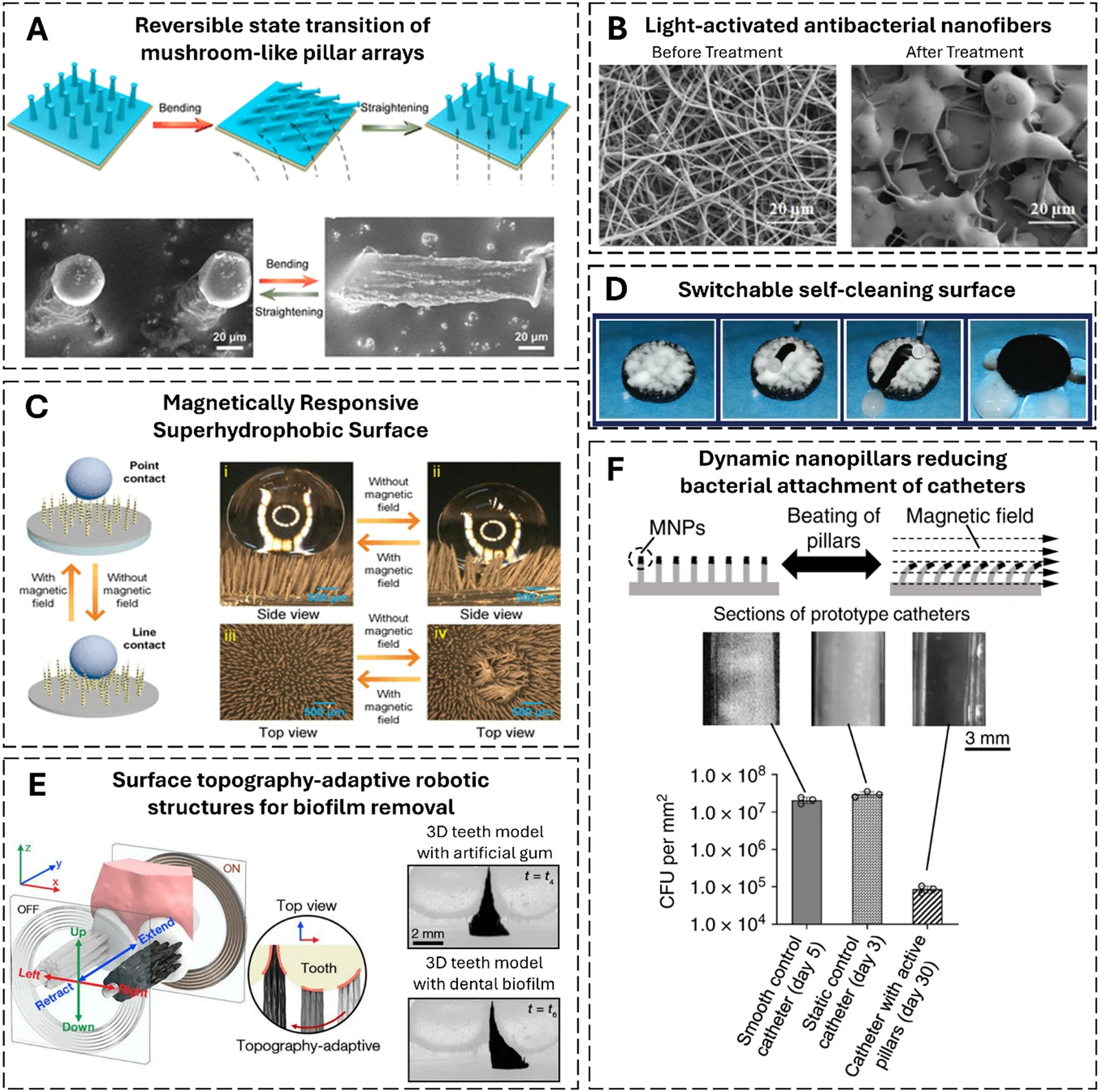
Next-generation dynamic surface topography. (A) Schematic and SEM images of reversible bending and stretching of mushroom-like pillars allowing for the creation of a superamphiphobic surface.^193^ (B) *meso*-Tetraphenylporphyrin (TPP)/polymethylmethacrylate (PMMA) nanofibers before and after a 20-hour light-emitting diode treatment.^194^ (C) Schematic and optical microscopy images showing magnetorheological elastomer micropillars transformed from collapsed morphology (water-adhesive state) to the fully upright position (water-repellent state).^195^ (D) Self-cleaning surfaces showing flour powder contamination preferentially adhering to water droplets and being successfully removed by fast drop slipping.^196^ (E) The forward electromagnet core guides the robotic superstructure bristles across the target surface, allowing for topographical adaptation, shape variation, and deep penetration into interdental space for biofilm removal.^192^ (F) Schematic of magnetic nanopillars (MNPs) moving due to magnetic field. The representative images show the complete blockage (visible white substance) of static control and flat control catheters, while the prototype catheters with MNPs remained clear, resulting in lower CFU per mm^2^.^191^ Images were reprinted (adapted) with permission.^193^ Wang *et al.*, (2019). Copyright 2019, American Chemical Society.^194^ Elashnikov *et al.*, (2016). Copyright 2016, Elsevier.^195^ Yang *et al.*, 2018. Copyright 2018, American Chemical Society.^196^ Děkanovský *et al.*, 2019. Copyright 2018, WILEY.^192^ Jun Oh *et al.*, (2024), licensed under CC.^191^ Gu *et al.*, (2020), licensed under CC.

Dynamic or ‘smart’ physical surface technologies represent a new frontier in antibacterial physical surface design. Unlike static topographies, these surfaces can adapt their physical properties, such as shape, size, stiffness, or wettability, in response to external stimuli like temperature, pH, light, or mechanical force (Fig. 3). This adaptability allows them to actively resist or disrupt microbial adhesion and biofilm formation in changing environments.^188–190^ For instance, thermoresponsive coatings can switch between hydrophilic and hydrophobic states, detaching adhered bacteria upon environmental changes. Similarly, shape-memory or magnetically actuated surfaces can generate mechanical forces that physically dislodge attached cells.^191^ In addition, the development of surface topography-adaptive robotic superstructures, capable of extending and retracting to dynamically adjust their shape, length, and stiffness, offers a promising approach for effective and targeted biofilm removal.^192^ These dynamic systems offer a promising strategy for prolonging surface efficacy and minimising microbial resistance development by introducing unpredictable and variable adhesion conditions.

## 4. The advantages and limitations of physical antibacterial approaches

Using surface topography to reduce bacterial adhesion and biofilm formation within the healthcare sector offers several significant advantages. Enhanced infection control is a primary benefit, as physical antibacterial strategies can substantially lower the risk of HAIs by creating surfaces inherently resistant to bacterial colonisation. This is especially crucial on medical devices and in hospitals and clinics, where stringent infection control is essential.^60,108,197^ Surface topographies could be strategically implemented on high-risk areas to passively reduce bacterial attachment and biofilm formation, thereby lowering the risk of pathogen transmission without reliance on reapplying chemical antimicrobial agents.

Physical surface strategies often exhibit broad-spectrum activity, being effective against a wide range of bacterial species, including both Gram-positive and Gram-negative bacteria, which is advantageous in diverse healthcare environments.^198,199^ Furthermore, because these strategies do not depend on specific molecular targets, bacteria are less likely to develop resistance, thereby mitigating the growing issue of AMR.^200–202^

Environmentally, physical approaches are more sustainable and eco-friendlier, as they do not involve the release or leaching of potentially harmful chemicals or metals over time.^108,203,204^ This makes them particularly suitable for applications where biocompatibility is crucial, such as in medical implants. With recent research supporting the use of topographies to control bacterial colonisation while supporting mammalian cell attachment and tissue integration.^106,156^

Lastly, the versatility of physical antibacterial techniques allows their application to a wide range of materials and devices, including metals, polymers, and ceramics, allowing for tailoring and engineering to suit specific applications and environments, and the creation of customised antibacterial solutions.^198^

Despite these advantages, there are also notable challenges in implementing these strategies. Manufacturing process considerations need to be made to ensure the pattern chosen is scalable, as techniques such as lithography, plasma treatments, or laser ablation might be intricate, time-consuming, and costly, which may limit large-scale applications.^205,206^ Integrating these technologies into existing healthcare infrastructure could be complex, necessitating substantial modifications to current practices and equipment. In turn, this can result in a high initial cost for implementation, due to the need for specialised equipment and processes, which can be a barrier to widespread adoption, particularly in resource-limited settings.

Due to the success of patterning surfaces to demonstrate antibacterial effects *in vitro*, various trials have explored their use *in vivo*. A review exploring nanopatterned titanium implants showed that effectiveness varied between preclinical studies and the *ex vivo* and *in vivo* experiments. Even though antibacterial activity was often seen in comparison to the control, it was noted that the patterns were not sufficient to target chronic bone infections.^207^ Patterning of surfaces should not be seen as a replacement for antibiotics or cleaning methods, but as an additional measure to provide bacterial colonisation and biofilm control. While physical approaches can be effective, their efficacy might be lower compared to strong chemical or antibiotic treatments, especially in environments with high bacterial loads or where other factors, such as nutrient availability, promote biofilm growth. Unlike chemical disinfectants, which kill bacteria quickly on contact, physical antibacterial surfaces often take time to work by preventing adhesion and colonisation over time, potentially limiting their effectiveness in situations requiring immediate bacterial eradication.

Moreover, *in vivo* applications, such as implanted medical devices, introduce additional challenges, such as micromotion at the tissue-implant interface, protein adsorption, and inflammatory responses that may progressively alter surface architecture. The durability of topographies in *in vivo* settings has yet to be extensively studied, but there is the risk of surface fragmentation, which could cause the loss of antibacterial activity over time and cytotoxicity to mammalian cells.^109,208^ Furthermore, surface topography may wear down due to mechanical abrasion, chemical exposure, or environmental factors, leading to reduced antibacterial effectiveness.^59^ While surface roughness can deter bacterial adhesion, excessively rough surfaces might trap debris or promote biofilm accumulation in surface irregularities, counteracting the intended antimicrobial effects. Ensuring consistent effectiveness may require regular maintenance and monitoring. In addition, new physical antibacterial technologies may face regulatory hurdles that can delay their market introduction, as they must undergo rigorous testing and approval processes to meet safety and efficacy standards.

While physical antibacterial approaches show great potential, challenges remain in optimising surface design for long-term effectiveness. Superhydrophobicity, for example, may degrade due to air layer depletion, reducing its antifouling capabilities. Potential future research could focus on developing robust, switchable surfaces that maintain their antibacterial properties under varying environmental conditions. Additionally, further studies are needed to refine the exact dimensions for maximal antibacterial efficacy while ensuring compatibility with biomedical and industrial applications and processes.

By harnessing physical antibacterial strategies, researchers can develop innovative and physical surface-modification solutions to mitigate bacterial contamination, offering promising advancements in healthcare.

## 5. Combination approaches

Recent research suggests that the most promising route for effective and durable bacterial control is the strategic combination of approaches,^209^ such as surface topography with chemical-based methods. While nanoscale or microscale surface structures can mechanically disrupt bacterial membranes, inhibit adhesion, and delay biofilm formation, these effects alone may be insufficient under complex biological or environmental conditions like healthcare settings. Therefore, coupled with approaches like antibiotics, chemical disinfectants, and antibacterial metals, can yield synergistic effects, enhancing both immediate bactericidal activity and long-term biofilm resistance. Physical structures can reduce bacterial load and delay colonisation, while potentially decreasing the dosage and frequency of chemical agents.^39,210–213^ This combined strategy not only improves performance^117^ but also helps lessen issues such as toxicity and the growing concern of AMR, ultimately decreasing our reliance on chemical-based interventions alone. Nonetheless, significant challenges remain, particularly concerning large-scale implementation, ensuring broad-spectrum efficacy, and preventing resistance that may emerge due to the inherently resilient nature of biofilms.^15,214^ More research is required, but this multimodal strategy represents a promising design principle for next-generation antimicrobials in healthcare settings.

## 6. Conclusions

Surface topography offers a promising, passive, and durable antibacterial strategy. By mimicking naturally occurring antibacterial surfaces, such as insect wings or plant leaves, engineered micro- and nano-scale structures can physically deter bacterial adhesion, proliferation, and biofilm formation. However, the effectiveness of these topographies is highly context-dependent, varying with bacterial species, surface chemistry, and environmental conditions. Different bacterial species exhibit distinct adhesion behaviours and biofilm-forming capacities depending on their shape, size, motility, and surface-sensing mechanisms. Consequently, surface designs must be tailored to specific applications, considering the dominant bacterial species likely to colonise a given medical device or environment.

Design strategies should also consider whether the intended outcome is antifouling (preventing attachment) or bactericidal (killing adhered bacteria). For instance, antifouling surfaces may be more beneficial for permanent implants to reduce biofilm formation associated with chronic infection, while bactericidal topographies may be more suitable for high-risk zones where sterilisation opportunities are limited. Although the antibacterial performance of such biomimetic surfaces has been well documented, the underlying mechanisms of action, particularly under dynamic, real-world conditions, remain an area of active research.

The successful implementation of topographical strategies also depends on practical and engineering considerations, including scalability, fabrication precision, long-term durability, and resistance to wear. Addressing these challenges requires systematic studies to develop a comprehensive library of well-characterised surface architectures, mapping their effects across bacterial species and environmental conditions. By first establishing a reliable experimental dataset, more effective ML and AI tools can be developed.

The current limiting factor of predictive models of antibacterial surface topographies is the lack of readily available, standardised, and high-quality datasets that capture the complexity of interfaces between bacteria and the patterned surface. Current research focuses narrowly on single outcome measures such as CFU counts or simple surface descriptors like roughness average. In addition, many studies are conducted under static, idealised laboratory conditions, often over 24 hours, which do not reflect physiologically relevant environments over time, leading to limited models. It is also important to note the continually evolving and complex nature of biological systems, and therefore, these datasets need to be continually updated to improve the model's accuracy. These predictive technologies in turn could accelerate surface design and screening, with the combination of resources helping guide the rational design of application-specific surfaces for medical devices and hospital infrastructure.

In summary, while physical antibacterial approaches offer substantial benefits, such as reduced antibiotic dependence, lower resistance risk, and sustained efficacy, they also face limitations related to manufacturing complexity, cost, and maintenance. Future advances are likely to emerge from dynamic surface topographies that can adapt, or combination approaches, where physical topographies are combined with chemical agents such as metal-based coatings or antibiotics to achieve synergistic, long-lasting protection. Translating these technologies into clinical practice will require continued interdisciplinary research focused on biocompatibility, long-term stability, and scalability. Ultimately, integrating topographical engineering with complementary antimicrobial strategies represents a critical frontier in developing safer, infection-resistant surfaces and healthcare environments.

## Conflicts of interest

All authors can confirm that we have no conflicts of interest to declare.

## Data Availability

All data supporting the findings of this study are contained within the article. This includes both original data generated in the course of the research and previously published data cited in the references.
